# A Qualitative Protocol to Examine Resilience Culture in Healthcare Teams during COVID-19

**DOI:** 10.3390/healthcare9091168

**Published:** 2021-09-06

**Authors:** John W. Ambrose, Diana M. Layne, Ken Catchpole, Heather Evans, Lynne S. Nemeth

**Affiliations:** 1 College of Nursing, The Medical University of South Carolina, Charleston, SC 29425, USA; layne@musc.edu (D.M.L.); nemethl@musc.edu (L.S.N.); 2 College of Medicine, The Medical University of South Carolina, Charleston, SC 29425, USA; catchpol@musc.edu (K.C.); evanshe@musc.edu (H.E.)

**Keywords:** resilience, healthcare team, qualitative research, immersion, crystallization, SEIPS framework, decision making, resilience engineering, teams, providers

## Abstract

Resilience allows teams to function at their optimal capacity and skill level in times of uncertainty. The SARS-CoV-2 (COVID-19) pandemic created a perfect opportunity to study resilience culture during a time of limited healthcare team experience, protocols, and specific personal protective equipment (PPE) needed. Little is known about healthcare team resilience as a phenomenon; existing definitions and empiric referents do not capture the nature of healthcare team resilience, as the traditional focus has been placed on individual resilience. This qualitative research protocol provides the rationale and methodology to examine this phenomenon and builds a bridge between resilience engineering and individual resilience. The sample is composed of healthcare team members from the US. This research may add to the body of knowledge on resilience culture in healthcare teams during the COVID-19 pandemic. This qualitative research protocol paper outlines the rationale, objective, methods, and ethical considerations entailed in this research.

## 1. Introduction

The SARS-CoV-2 (COVID-19) pandemic created a perfect opportunity to study resilience culture during a time of limited healthcare team experience, protocols, and specific personal protective equipment (PPE) needed [1,2,3]. The concept of resilience has been described in the literature as “bouncing back” and “bouncing forward [4]. Healthcare systems continue to have a critical need to protect their employees and reduce further viral transmission from COVID-19 and its variants in the US. From January 2020 until 4 September 2021, over 39.8 million COVID-19 infections and 644,592 deaths were reported [5].

Resilience engineering provides the operational definition of resilience for this study protocol; namely, as the ability of a healthcare team to adjust its functioning prior to, during, or following acute changes and disturbances for the team to perform its duties in both anticipated and unanticipated conditions [6]. Among healthcare workers, over a third of nursing-related healthcare providers were hospitalized due to COVID-19 between March and May of 2020 [7]. More than 7000 deaths of healthcare workers were reported worldwide. The US alone reported over 2900 deaths of healthcare workers (e.g., nurses, nurse practitioners, doctors, environmental service providers, respiratory therapists, etc.) [8,9]. 

Infections among healthcare team members may reduce the number of healthcare providers available to deliver safe, quality care. This creates unsafe staffing environments for healthcare teams and the patients they care for. For example, nurses without the knowledge and skills needed to meet the standard of care may be redeployed to other clinical areas within their hospitals. Resilience among healthcare team members is required to adapt to new clinical environments due to rapidly changing information from the Centers for Disease Control (CDC), and sometimes limited PPE. 

### 1.1. Understanding Resilience of Healthcare Teams

Resilience has been described in individuals within healthcare teams. Little is known about how healthcare teams become resilient in the face of a pandemic. Herein, we make the distinction between the idea of team as a group of discrete individuals and a team as a collective entity. Understanding healthcare team resilience during COVID-19 is important because health systems must continually (1) ensure the safety of healthcare team members, (2) ensure the adaptive capacity of the healthcare system, (3) meet clinical staffing needs, and (4) educate healthcare teams about new practice guidelines (e.g., PPE protocols, new procedures and use of technology (telemedicine, UV disinfection, etc.). 

### 1.2. Existing Protocols Are Insufficient

Recent publications suggest the need to specifically increase resilience during the pandemic [10,11,12]; however, there is a lack of prospective research specific to healthcare teams and the necessary protocols needed to maintain safety and prevent the spread of viral infection to others [13]. For example, protocols are needed for use of ultraviolet disinfection, mechanical ventilation, safe staffing patterns, isolation precautions and surgical interventions [13,14,15,16,17].

A recent concept analysis of resilience in healthcare teams suggested that resilience: (a) has a reciprocal relationship with social support that can help individuals with their ability to adjust and move on; (b) is associated with the decreased incidence of co-morbid disorders such as posttraumatic stress disorder (PTSD), depression, and dysfunctional anxiety; and (c) may be enhanced by flexible and trustworthy communication among healthcare teams [4]. The ongoing pandemic provides a unique opportunity to better understand the factors that impact resilience within healthcare teams to develop future protocols.

### 1.3. Critical Need to Be Prepared for Ongoing and Future Pandemics 

COVID-19 created unprecedented change in the healthcare environment compared to past Influenza A, SARS, MERS and Ebola outbreaks [17,18,19,20]. Unlike the other viruses, COVID-19 is highly contagious; has a longer incubation time and there are no well-established standardized treatments [17,18,19,20]. Healthcare teams were unknowingly exposed to asymptomatic carriers in the hospital and in the community. A lack of PPE, limited manpower, and dwindling routine supplies, from interruptions in the supply chain, created environments with an abundance of stress, fatigue, and fear [9,21]. 

The PPE that was available limited the healthcare team’s physical senses of sight (fog), hearing (muffled sounds from multiple mask layers), touch (gloves), and smell (masks). These physical limitations challenged the everyday physical assessment of patients and created barriers to routine team functions. The need for social distancing to prevent the spread of infection may impact the healthcare teams’ ability to socialize and support one another. The added stress and risk of PTSD threaten the team’s ability to maintain its routine function [21]. 

The discovery of key indicators of healthcare team resilience may identify strategies to improve teamwork within organizations, improve the team’s overall physical and mental health, and promote effective communication [21,22,23,24,25,26]. 

Accordingly, this research seeks to (1) explain resilience culture in healthcare teams, (2) identify key indicators and barriers to resilience, (3) describe requirements of healthcare teams, to pivot as a team, to increase their support for one another and the organization. 

The novelty of this research protocol is that it specifically focuses on resilience culture in teams. PPE and team function are used as exemplars to reflect the components of individual, team, and system resilience. 

## 2. Materials and Methods

### 2.1. Qualitative Study Design: General Approach and Objectives

Constructivist inquiry will be used to understand the human construct of resilience culture through the experiences of the participants (healthcare team personnel: e.g., nurses, doctors, administrators, etc.) [27]. This paradigm is appropriate to gain wisdom from the stories of the informants [27]. We acknowledge participants possess varied experiences [28]. 

Tenets of Bradley’s approach to qualitative analysis for health services research is best suited for this qualitative study. These include (1) immersion into the interview data to understand its scope and context of participant experiences and (2) coding to organize the interview data [28]. An inductive and deductive descriptive approach will be employed. The codes will be refined through constant comparison, until no new concepts emerge from the data and *theoretical saturation* has been achieved [27,28,29,30,31]. Application of the final code structure will be reviewed by an expert qualitative researcher on the team to describe themes surrounding (1) resilience culture in healthcare teams, (2) key indicators and barriers to resilience, (3) the requirements of healthcare teams to pivot as a team to increase support for one another and the organization.

### 2.2. Guiding Framework for Research

#### 2.2.1. Adapted Conceptual Model

**Figure 1:**
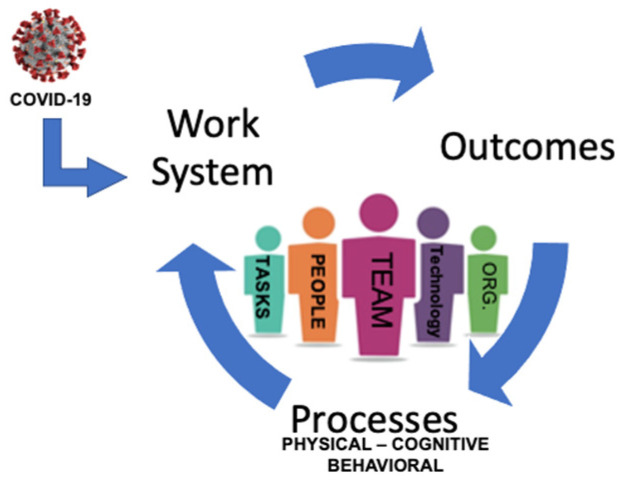
Advanced Decision Making and Systems Engineering for Patient Safety Adapted Model.

An adapted conceptual model was developed for this research, which combines the The Systems Engineering for Patint Safety (SEIPS) framework [32] version 1.0 and the Advanced Team Decision-Making model (ADM) [33] (see Figure 1 below). When uncertainty is experienced, the adapted model illustrates (1) the healthcare team as a discrete entity at its center (2) the dynamic interchange between the work system, (3) the healthcare team’s physical, cognitive, and behavioral processes, and (4) the outcomes the healthcare team achieves. 

The SEIPS framework is based on systems; their inputs, processes, and outcomes [32]. When combined with the Advanced Decision-Making model [33], the adapted model provides a useful illustration to understand resilience culture in healthcare teams. The Advanced Decision-Making Model focuses on the team as a discrete entity, capable of making its own decisions, and thus removes the focus from the individual [31]. The model incorporates four interrelated elements: (1) team resources, (2) team identity, (3) team cognition, and (4) team metacognition [33]. 

The strength of the adapted model may be its applicability to any context of disruption or state of uncertainty. It acknowledges the synergy between the work system, teams of professionals, available technologies and tools, the organization, environment, tasks, processes within the system (patient care and other routine team functions), and the outcomes achieved (quality metrics, patient safety, staff satisfaction, retention). 

#### 2.2.2. Healthcare Team Resilience 

Resilience culture in healthcare teams is influenced by its people, available resources (supplies, manpower, technology), daily work tasks (assessment, planning, implementation, evaluation, and organizational culture). The outcomes achieved by the healthcare team provide feedback which informs the future actions of the healthcare team [34,35,36,37].

On a granular level, a healthcare team member who is judged by the team to lack resilience based on outcomes; or other metrics (real or perceived), may lead to a healthcare team culture of blame. A paradigmatic pivot to understand healthcare team as a collective entity and resilience as a favorable team quality, shifts the focus from the individuals who comprise a healthcare team to the organizational systems and processes that support the healthcare team. A shift from an organizational culture of blame to a culture of collegiality and support benefits patients and healthcare personnel [38].

### 2.3. Setting(s)

This study protocol includes hospitals across the Unites States in the Northeast, South, Midwest, and West Coast. The emergency room, intensive care unit, COVID-ICU, COVID-floor, gastroenterology/endoscopy, operating room, post anesthesia recovery room, holding area, clerical/scheduling, and inpatient medical and surgical units would be included. 

### 2.4. Sampling Strategy, Size, and Eligibility

#### 2.4.1. Sampling, Inclusion, Exclusion

A purposive sample of interprofessional healthcare team members including nurses, physicians, patient technologists, surgeons, surgical technologists, proceduralists, nurse anesthesiologists, anesthesiologists, administration manager/director, senior administrator DON, CMO, support staff, environmental service staff, who were employed between January 2020 and December 2020, will be invited to participate. 

Purposive sampling will be used to identify healthcare team participants to provide data, in the form of their stories, related to the phenomenon of interest. In this case, stories of resilience during COVID-19 would be best told by those who worked in varied interprofessional healthcare team roles during the pandemic. This sampling method helps ensure that the phenomenon (resilience culture) is informed by the individuals most likely to offer valuable perspectives from different points of view. To represent the fullest picture of interprofessional experience, intention will be paid to recruit across the divergent geographic participant groups through personal contacts and social media [27,28,29,30]. 

##### Inclusion Criteria

Members of United States healthcare teams, including women and men ages 20 and above with a personal email address, access to the internet, the ability to click a hypertext link and navigate a website, and the ability to understand the English language will be included. These individuals must have been employed in the emergency room, intensive care unit, COVID-ICU, COVID-floor, gastroenterology/endoscopy, operating room, post anesthesia recovery room, holding area, clerical/scheduling, inpatient medical and or surgical areas between the months of January 2020–December 2020.

##### Exclusion Criteria

Healthcare team members who do not meet the inclusion criteria, who cannot physically or emotionally tolerate an interview will be excluded.

#### 2.4.2. Sample Size

Approximately 10 individuals will be recruited from the primary study site. Snowball sampling will be used to reach approximately 20–30 additional eligible participants across the United States using social media and personal contacts for a total sample size of approximately 40. Recruiting will continue until data saturation is achieved. Data saturation is defined as the point at which no new themes emerge from participant interviews [27,28,29,30,31].

#### 2.4.3. Recruitment

Individuals who meet the inclusion criteria will be invited by the principal investigator and research team members to participate through word of mouth and the use of fliers and social media posts. Fliers will be placed by a proxy at central locations on the study site hospital and college campuses and will also be displayed electronically on Facebook^TM^, Menlo Park, CA, USA, Twitter^TM^, San Francisco, CA, USA and Instagram^TM^, San Francisco, CA, USA. Potential study participants will be sent a link to a REDCap^TM^, Nashville, TN, USA, screening survey that will explain the purpose of the study and verify their eligibility.

Informants who participate in the interview will receive the code for an Amazon gift card after the interview is completed. This incentive will be funded by the primary investigator. Interviews will be scheduled on a date and time that is convenient for the participant to decrease chances of attrition.

Consent would be obtained when the participant “clicks-through” on REDCap^TM^. A video/audio interview will be scheduled with the participant on Microsoft^®^ Teams. The deidentified interview data will be uploaded to NVivo^TM^ for transcription. The transcribed interviews will be compared to the original audio recording and edited to reflect the real-time comments from the participant. The informants’ demographic data will be collected via REDCap^TM^ and will include their primary practice area, deployment status, current role/position, years of experience, geographic location, age, and race. 

A study identification number will be randomly assigned by MS Excel^TM^, Redmond, WA, USA, and used to deidentify each participant to protect their privacy. Informants will be contacted to schedule a video/audio interview on Microsoft^®^ Teams. Each semi-structured interview will be conducted by a single interviewer, which is preferred in this type of research [28,29,30,31]. 

The principal investigator, experienced in nursing research and having completed a fellowship in clinical and translational research ethics, will conduct the interviews. The interviewer will debrief with an expert qualitative researcher within the team to continuously during data collection and throughout the final analysis. Each interview will last between 30 to 45 min. Reflective memos will be written to highlight observations and perceptions of related to the interviews at the conclusion of each session.

The interview guide is derived from SEIPS and ADM models to explore the informants’ knowledge of resilience culture and decisions made during COVID-19. These questions were pilot tested with the first three interviews and no changes were needed. The questions connect the individual to the team and the team to the organization, with the emphasis on the team [39].

### 2.5. Data Monitoring, Safety and Analysis

#### 2.5.1. Data Safety and Monitoring

Both the demographic and interview data will be secured on web-based platforms to protect participants’ privacy and confidentiality. The demographic data obtained from the eligibility screening questionnaire built on REDCap^TM^ will be stored securely on REDCap^TM^. This web-based application requires a two-step authentication process. The quality and completeness of the data will be monitored daily to ensure all fields have been completed. Incomplete and missing data will be removed from the data set. 

Recorded interviews with video and audio would take place on Microsoft^®^ Teams, Redmond, WA, USA to ensure social distancing and enhance convenience for the participants. The semi-structured interview guide is shown in Table 1. All interviews will be deidentified and given a random identification number generated by MS Excel^TM^. Interviews will be uploaded to and transcribed via qualitative research software NVivo^TM^ release 1.4 for MAC, (QSR International, Victoria, Australia). NVivo^TM^ ensures data is secure by the encryption of the transcribed data as it is obtained and transmitted to the website. The PI of the research team will have sole access to and control over data. 

Codebooks, eIRB Log, and other study records will be stored on Box^TM^. End-user devices will be kept under control of each member of the research team. Additionally, QSR International (the makers of NVivo^TM^) uses Microsoft Azure cloud services, which aligns with the regulation for General Data Protection Regulation (GDPR) COM/2012/010. In addition, NVivo^TM^ Transcription is HIPAA (Health Insurance Portability and Accountability Act 1996) compliant [40]. Both a) health data protection standards established by HIPPA and b) administrative, physical, and technical safeguards put into place by QSR International will ensure compliance with HIPAA regulations [40].

#### 2.5.2. Data Analysis

Demographic data will be input into SPSS^®^ IBM, Chicago, IL, USA, Statistics for MAC, version 25. Qualitative data will be uploaded to NVivo^TM^ software, release 1.4 for MAC (QSR International, Pty, Victoria, Australia) for qualitative analysis. 

Our qualitative analysis will employ two approaches: (1) Bradley’s qualitative research for health care (inductive and deductive strategy to develop taxonomies and themes) [28] and (2) Gale’s seven stage framework method (transcription, familiarization with the interview, coding, developing a working analytical framework, applying the analytical framework, charting data into interview framework matrix, interpreting the data) [29]. Each interview will be reviewed to ensure accuracy. All transcripts will be read by the researcher first, and then coded in a line-by-line fashion using open coding [27]. Inductive/deductive “ground-up” codes will be generated from each informant’s unique experience [27]. Characteristic words and phrases from each informant allow insight into the sample group of informants as a whole [27]. Deductively, participant characteristics and setting codes will be created from each informant’s data. 

Our multidisciplinary team (human factors expert, nurse scientists, surgeon with qualitative expertise) will re-review all the data to apply the existing code structure. Each team member will record the way they arrive at their personal findings. In a final meeting, through immersion and crystallization [27,41]. Immersion crystallization is a style of qualitative analysis that involves cycles of data review, reflection and intuitive insight which culminates in a reportable interpretation [41]. The group will reconvene, review any discrepancies, and come to consensus for a final code structure [27,28,29,30,31,41,42]. Both independent review and group discourse will strengthen our analyses. 

Demographic data will be analyzed by the PI using IBM SPSS Statistics for MAC, version 25. In addition to a written narrative, nominal demographic data will be presented in a bar graph. Descriptive statistics (mean, standard deviation) will be calculated for the continuous variables of age and years of experience. 

### 2.6. Protectin of Human Subjects

#### 2.6.1. Risk to Participants

Participants may experience emotional discomfort as they recount their experiences. Psychological support through proper channels will be encouraged if the informant desires. This study presents no greater than minimal risk to participants and meets exempt status per regulatory criteria established by 45 CFR 46.104 and 21 CFR 56.104. Participant’s employment status will not be affected should they decline to participate in or withdraw from this study. There is a very low risk for a breach in the participant’s confidentiality and privacy. 

#### 2.6.2. Adequacy of Protection against Risks

This research would be conducted in an ethical manner with respect to human subjects and in accordance with the study site policy. To ensure that potential negative consequences from a breach of confidentiality are avoided, participants will be sent a secure e-mail. A twelfth grade reading level was chosen to reflect the minimum education required to work in the healthcare setting in the US. Researchers who choose to replicate this study may decide which reading level fits their target population. The email will present an overview and purpose of the study and detail the risks and benefits to the participant. The email will also include an explicit statement of the potential informant’s right to refuse to participate. 

The present proposed study has been approved by the MUSC IRB and is registered under ID Pro00100917.

## 3. Discussion 

This is a protocol paper intended to describe research protocol rather than show results. The results from this study may further define resilience culture within healthcare teams. This is a key step in the development of an empiric instrument to assess healthcare team resilience culture and interventions to support teams as discreet entities and not the sum of their constituent parts (individual team members).

## 4. Conclusions

The data will provide accounts of resilience, how it is perceived and how it manifests in teams during the COVID-19 pandemic. Resilience is essential for healthcare teams to “bounce back” and “move forward” to provide and maintain safe patient care.

## Figures and Tables

**Table 1:** Interview Guide.

Domain Type	Interview Content Probes: Resilience Culture & Decision Making During COVID-19
**Experience**	Tell me what it was like for you? How did the ongoing pandemic change the team culture and decision making?
**Basis**	How were decisions selected and others rejected?
**Aiding**	Tell me about how you think critical decisions worked out • If not so well, what additional knowledge, training or support might have helped the team? • What feedback would you give administration on how to enhance safety?
**Time**	How much time pressure was involved in making decisions?
**Knowledge**	Describe a day or experience related to working in the early days of the COVID-19 pandemic that stood out for you. • How did you keep the team safe? • What PPE changes did you make, procedures for donning and doffing PE, caring for your N95? • How did your self-care practices change? • What COVID-19 testing approaches were used? • After exposure to COVID-19, what hospital changes were made to ensure the safety of the team?
**Situational Assessment**	What changes did you face related to your specific role on the team?

## References

[1] Cohen J., van der Meulen Rodgers Y. (2020). Contributing factors to personal protective equipment shortages during the COVID-19 pandemic. Prev. Med..

[2] Botti C., Lusetti F., Castellucci A., Costantini M., Ghidini A. (2020). Safe tracheotomy for patients with COVID-19. Am. J. Otolaryngol..

[3] Lancaster E.M., Sosa J.A., Sammann A., Pierce L., Shen W., Conte M.C., Wick E.C. (2020). Rapid Response of an Academic Surgical Department to the COVID-19 Pandemic: Implications for Patients, Surgeons, and the Community. J. Am. Coll. Surg..

[4] Ambrose J.W., Nemeth L.S., Layne D.M. (2021). A Systematic Concept Analysis of Healthcare Team Resilience in Times of Disaster and Pandemic. Nurs. Forum.

[5] Johns Hopkins Corona Virus Resource Centre Coronavirus Disease. https://coronavirus.jhu.edu/map.htmlt.

[6] Hollnagel E., Pariès J., Wreathall J. (2011). Resilience Engineering in Practice A Guidebook.

[7] Kambhampati A.K., O’Halloran A.C., Whitaker M., Magill S.S., Chea N., Chai S.J., Kirley P.D., Herlihy R.K., Kawasaki B., Meek J. (2020). COVID-19–Associated Hospitalizations Among Health Care Personnel—COVID-NET, 13 States, 1 March–31 May 2020. MMWR Morb. Mortal. Wkly. Rep..

[8] Jewett C., Lewis R., Bailey M. (2020). More Than 2900 Health Care Workers Died This Year—And the Government Barely Kept Track. https://khn.org/news/article/more-than-2900-health-care-workers-died-this-year-and-the-government-barely-kept-track/.

[9] Cockburn S. (2020). Global: Amnesty Analysis Reveals over 7000 Health Workers Have Died from COVID-19. https://www.amnesty.org/en/latest/news/2020/09/amnesty-analysis-7000-health-workers-have-died-from-covid19/.

[10] Hughes M.M., Groenewold M.R., Lessem S.E., Xu K., Ussery E.N., Wiegand R.E., Qin X., Do T., Thomas D., Tsai S. (2020). Update: Characteristics of Health Care Personnel with COVID-19—United States, 12 February–16 July 2020. MMWR Morb. Mortal. Wkly. Rep..

[11] Frias C.E., Cuzco C., Martín C.F., Perez-Ortega S., López J.A.T., Lombraña M. (2020). Resilience and Emotional Support in Health Care Professionals During the COVID-19 Pandemic. J. Psychosoc. Nurs. Ment. Health Serv..

[12] O’Sullivan B., Leader J., Couch D., Purnell J. (2020). Rural Pandemic Preparedness: The Risk, Resilience and Response Required of Primary Healthcare. Health Policy Politi Sante.

[13] Mermel L.A. (2020). Disposition of patients with coronavirus disease 2019 (COVID-19) whose respiratory specimens remain positive for severe acute respiratory coronavirus virus 2 (SARS-CoV-2) by polymerase chain reaction assay (PCR). Infect Control Hosp. Epidemiol..

[14] Pilar A., Gravel S.B., Croke J., Soliman H., Chung P., Wong R.K.S. (2021). Coronavirus Disease 2019’s (COVID-19’s) Silver Lining—Through the Eyes of Radiation Oncology Fellows. Adv. Radiat. Oncol..

[15] Raeiszadeh M., Adeli B. (2020). A Critical Review on Ultraviolet Disinfection Systems against COVID-19 Outbreak: Applicability, Validation, and Safety Considerations. ACS Photon.

[16] Dar M., Swamy L., Gavin D., Theodore A. (2021). Mechanical-Ventilation Supply and Options for the COVID-19 Pandemic. Leveraging All Available Resources for a Limited Resource in a Crisis. Ann. Am. Thorac. Soc..

[17] Lasater K.B., Aiken L.H., Sloane D.M., French R., Martin B., Reneau K., Alexander M., McHugh M.D. (2020). Chronic hospital nurse understaffing meets COVID-19: An observational study. BMJ Qual. Saf..

[18] Liang S.T., Liang L.T., Rosen J.M. (2021). COVID-19: A comparison to the 1918 influenza and how we can defeat it. Postgrad. Med. J..

[19] Krishnan L., Ogunwole S.M., Cooper L.A. (2020). Historical Insights on Coronavirus Disease 2019 (COVID-19), the 1918 Influenza Pandemic, and Racial Disparities: Illuminating a Path Forward. Ann. Intern. Med..

[20] Lal A., Ashworth H.C., Dada S., Hoemeke L., Tambo E. (2020). Optimizing Pandemic Preparedness and Response through Health Information Systems: Lessons Learned from Ebola to COVID-19. Disaster Med. Public Health Prep..

[21] Carmassi C., Foghi C., Dell’Oste V., Cordone A., Bertelloni C.A., Bui E., Dell’Osso L. (2020). PTSD symptoms in healthcare workers facing the three coronavirus outbreaks: What can we expect after the COVID-19 pandemic. Psychiatry Res..

[22] Patterson G.E., McIntyre K.M., Clough H.E., Rushton J. (2021). Societal Impacts of Pandemics: Comparing COVID-19 With History to Focus Our Response. Front. Public Health.

[23] Albott C.S., Wozniak J.R., McGlinch B.P., Wall M.H., Gold B.S., Vinogradov S. (2020). Battle Buddies: Rapid Deployment of a Psychological Resilience Intervention for Health Care Workers During the COVID-19 Pandemic. Anesth. Analg..

[24] Barzilay R., Moore T.M., Greenberg D.M., DiDomenico G.E., Brown L.A., White L.K., Gur R.C., Gur R.E. (2020). Resilience, COVID-19-related stress, anxiety and depression during the pandemic in a large population enriched for healthcare providers. Transl. Psychiatry.

[25] Donnelly P.D., Davidson M., Dunlop N., McGale M., Milligan E., Worrall M., Wylie J., Kidson C. (2020). Well-Being during Coronavirus Disease 2019: A PICU Practical Perspective. Pediatr. Crit. Care Med. J. Soc. Crit. Care Med. World Fed. Pediatr. Intensive Crit. Care Soc..

[26] Hartwig A., Clarke S., Johnson S., Willis S. (2020). Workplace team resilience: A systematic review and conceptual development. Organ. Psychol. Rev..

[27] Crabtree B.F., Miller W.L. (1999). Clinical Research. Doing Qualitative Research.

[28] Bradley E.H., Curry L.A., Devers K.J. (2007). Qualitative Data Analysis for Health Services Research: Developing Taxonomy, Themes, and Theory. Health Serv. Res..

[29] Gale N.K., Heath G., Cameron E., Rashid S., Redwood S. (2013). Using the framework method for the analysis of qualitative data in multi-disciplinary health research. BMC Med. Res. Methodol..

[30] Lincoln Y., Guba E. (1985). Naturalistic Inquiry.

[31] Vaismoradi M., Turunen H., Bondas T. (2013). Content analysis and thematic analysis: Implications for conducting a qualitative descriptive study: Qualitative descriptive study. Nurs. Health Sci..

[32] Carayon P., Hundt A.S., Karsh B.-T., Gurses A.P., Alvarado C.J., Smith M., Brennan P.F. (2006). Work system design for patient safety: The SEIPS model. Qual. Saf. Health Care.

[33] Klein G.A., Sullivan J. (2001). Sources of Power: How People Make Decisions. Leadersh. Manag. Eng..

[34] Holden R.J., Carayon P. (2021). SEIPS 101 and seven simple SEIPS tools. BMJ Qual. Saf..

[35] Bandura A., Freeman W.H., Lightsey R. (1999). Self-Efficacy: The Exercise of Control. J. Cogn. Psychother..

[36] Bandura A. (1989). Human Agency in Social Cognitive Theory. Am. Psychol..

[37] Bandura A. (2001). Social Cognitive Theory of Mass Communication. Media Psychol..

[38] Brborović O., Brborović H., Nola I.A., Milošević M. (2019). Culture of Blame—An Ongoing Burden for Doctors and Patient Safety. Int. J. Environ. Res. Public Health.

[39] Klein G.A., Calderwood R., MacGregor D. (1989). Critical decision method for eliciting knowledge. IEEE Trans. Syst. Man Cybern..

[40] di Gregorio S. (2019). Transcrioption: More Than Just Words. https://www.qsrinternational.com/nvivo-qualitative-data-analysis-software/resources/blog/transcription-more-than-just-words#:~:text=It’s%20secure%20and%20private,This%20is%20fully%20GDPR%20compliant.

[41] Borkan J. (1999). Immersion/Crystallization. Doing Qualitative Research.

[42] Williams M., Moser T. (2019). The Art of Coding and Thematic Exploration in Qualitative Research. Int. Manag. Rev..

